# Sport participation and healthy aging among older adults: a social ecological analysis of individual, contextual, and policy mechanisms

**DOI:** 10.3389/fpubh.2026.1890213

**Published:** 2026-09-17

**Authors:** Lide Su, Agudamu, Bo Long

**Affiliations:** 1 College of Physical Education, Hunan Normal University, Changsha, China; 2 Graduate School of Social Welfare, Sungkyunkwan University, Seoul, Republic of Korea

**Keywords:** community environment, healthy aging, older adults, public health, sport participation

## Abstract

**Introduction:**

Population aging has made healthy aging a key public health priority. This study examined the association between sport participation and healthy aging among older adults and explored its social ecological mechanisms.

**Methods:**

Data from 6,482 adults aged 60 years and above were analyzed. Healthy aging was measured using a multidimensional index covering physical, psychological, cognitive, social and functional domains. Multivariable regression, mediation analysis, moderation analysis, subgroup analysis and robustness checks were conducted.

**Results:**

Sport participation was positively associated with healthy aging after adjustment for demographic, socioeconomic, health-related and ecological factors. The association showed a dose-response pattern and was partly mediated by physical function, psychological resources, social support and community activity environment. Community resources strengthened this association, while stronger effects were observed among rural, low-income, less educated, older-old and living-alone groups.

**Discussion:**

The results suggest that sport participation is closely related to healthy aging through individual and social ecological pathways. Age-friendly sport environments and targeted community support may help promote healthy aging among vulnerable older adults.

## 1 Introduction

Population aging has become one of the most consequential public health issues facing contemporary societies (1). With the continuous extension of life expectancy and the rapid growth of the older population, the focus of aging research has gradually shifted from survival in later life to the quality, function, dignity and social participation associated with longer lives. For older adults, health is no longer limited to the absence of diagnosed disease (2). It is reflected more concretely in the ability to maintain physical function, preserve psychological well-being, participate in social life, manage chronic conditions and sustain a relatively independent daily life (3). This multidimensional understanding is consistent with the concept of healthy aging, which emphasizes functional ability and the interaction between individual capacities and supportive environments (4). In this sense, improving healthy aging is not only a medical issue, but also a broader public health task involving behavior, community resources, social relationships and institutional support.

Physical activity is widely regarded as a modifiable behavioral factor closely related to health in later life. For older adults, regular participation in sport and exercise may help maintain cardiopulmonary function, muscle strength, balance, mobility and metabolic stability, while also contributing to emotional regulation, cognitive maintenance and social connection (5). Compared with general daily physical activity, sport participation usually has clearer behavioral intention, relatively stable frequency, recognizable activity forms and stronger social or spatial attributes. Activities such as walking groups, tai chi, square dancing, table tennis, fitness exercises and community-based sports programs are embedded in specific social settings and often involve interaction with peers, access to public spaces and reliance on community organization (6). Therefore, the health significance of sport participation among older adults lies not only in energy expenditure or physical training (7), but also in its potential to connect bodily function, psychological resources and social participation.

Existing studies have provided substantial evidence that physical activity is associated with lower risks of chronic disease, disability, depressive symptoms, cognitive decline and premature mortality among older adults (8). However, several limitations remain in the current literature. Many studies focus on single health outcomes, such as self-rated health, depressive symptoms or activities of daily living, which makes it difficult to capture the overall level of healthy aging (9). Some studies measure physical activity in a relatively simplified way, often distinguishing only between participation and non-participation, without sufficient attention to frequency, intensity, duration and organizational form. More importantly, the pathway through which sport participation contributes to healthy aging has not been adequately examined (10). Older adults’ participation in sport is shaped by health status, family support, neighborhood relationships, built environment, public service provision and social norms (11). Ignoring these contextual conditions may lead to an incomplete understanding of why some older adults benefit more from sport participation than others.

Gender differences in older adults’ sport participation should be understood within broader social and institutional contexts. Although older women and men may share similar motivations for maintaining health and social engagement, their opportunities to participate in sport are often shaped by different life-course experiences and social responsibilities. In many societies, including China, older women have historically undertaken substantial unpaid family care responsibilities, including childcare support, household management and caregiving for dependent family members. These responsibilities may reduce discretionary time available for regular exercise and influence the continuity of sport participation. At the micro level, gender differences may emerge through variations in physical capability, health perception and exercise confidence. Older women often experience higher rates of chronic conditions, functional limitations and fear of injury, which may influence their willingness to engage in certain forms of physical activity. At the same time, culturally familiar activities such as Tai Chi, walking groups and square dancing may provide relatively accessible forms of participation by reducing psychological and physical barriers.

The social ecological perspective offers a useful framework for understanding this issue (12). This perspective places individual health behavior within multiple interacting layers, including personal characteristics, family resources, interpersonal networks, community environments and broader institutional conditions. Applied to older adults’ sport participation, this means that participation is influenced by physical capacity, health literacy, exercise self-efficacy, family encouragement, peer companionship, neighborhood safety (13), accessibility of sports facilities, green spaces and the availability of age-friendly public services. These factors may not only affect whether older adults engage in sport, but also shape the extent to which sport participation can be transformed into better health outcomes (14). For example, an older adult living in a safe community with accessible exercise spaces and active peer groups may find it easier to maintain regular participation and gain psychological and social benefits from exercise (15). In contrast, limited mobility, lack of companionship, poor facility access or weak community organization may reduce both participation opportunities and health returns.

From a public health perspective, the relationship between sport participation and healthy aging also involves the issue of health equity (16). Older adults differ markedly in socioeconomic status, education, residential location, family structure and access to community resources (17). These differences may produce unequal opportunities for physical activity and unequal benefits from participation. Urban older adults may have better access to parks, walking spaces, sports facilities and organized community activities, while rural older adults may face more constraints in public service supply and activity organization (18). Older adults living alone or with limited family support may experience greater barriers to sustained participation. Low-income or less educated groups may have weaker health literacy and fewer opportunities to participate in structured exercise programs (19). Therefore, examining the social ecological mechanism of sport participation is necessary for identifying not only average health effects, but also the conditions under which sport participation can contribute to more inclusive healthy aging.

In this study, sport participation refers to regular engagement in structured or semi-structured physical activities that involve intentional exercise behavior, repeated participation and certain forms of social or organizational connection. This conceptualization differentiates sport participation from general physical activity in daily life. Although daily activities such as household work, commuting and occasional walking contribute to energy expenditure and mobility maintenance, they often lack stable participation patterns and organized social contexts. Sport participation among older adults represents a more sustained behavioral practice that combines physical training, social interaction and psychological engagement.

The meaning of sport participation among older adults is also shaped by cultural and social contexts. In Western contexts, sport participation has often been discussed through the perspectives of fitness improvement, recreational activity and individual health behavior. In China, however, many common forms of older adults’ sport participation are closely connected with community life and traditional health concepts. Practices such as Tai Chi, square dancing, walking groups and community fitness activities combine physical movement with social interaction, collective participation and neighborhood communication. Their health value therefore extends beyond physiological improvement and involves the reconstruction of social connections and everyday routines after retirement.

From this perspective, sport participation can be understood as a socially embedded health behavior within the process of healthy aging. Its effects depend not only on individual willingness and physical capacity, but also on whether older adults have access to supportive social networks, suitable community spaces and public services. The social ecological framework provides a useful explanation for this process by linking individual exercise behavior with interpersonal support, community environments and institutional resources. Through these interconnected pathways, sport participation may contribute to the maintenance of functional ability, improvement of psychological well-being and enhancement of social participation, which together constitute important dimensions of healthy aging.

## 2 Materials and methods

### 2.1 Study architecture and social ecological logic

This study used an observational quantitative design to examine the association between sport participation and healthy aging among older adults (20). The analytical framework was guided by the social ecological model, which assumes that health-related behavior in later life is shaped by interacting factors at the individual, family, interpersonal, community and service levels. In this study, sport participation was treated as the core exposure, healthy aging as the primary outcome, and social ecological conditions as explanatory mechanisms that may transmit or condition the effect of sport participation on healthy aging.

The empirical logic followed three connected steps. The baseline analysis estimated whether older adults who participated in sport showed higher levels of healthy aging after adjusting for demographic, socioeconomic, lifestyle and health-related confounders. The mechanism analysis examined whether physical function, psychological resources, social support and community activity conditions explained part of this association. The heterogeneity analysis tested whether the association varied by residence, gender, age group, education, income and living arrangement. This structure allowed the study to move from average association to mechanism identification and then to health equity interpretation.

The conceptual model can be expressed as follows.


$${\mathrm{HA}}_{i}=f({\mathrm{SP}}_{i},M_{i},E_{i},X_{i})$$

In this formula, $HA_{i}$ denotes the healthy aging level of older adult i , $SP_{i}$ denotes sport participation, $M_{i}$ represents mediating variables such as social support or psychological well-being, $E_{i}$ represents ecological conditions such as community facility accessibility and neighborhood environment, and $X_{i}$ denotes covariates.

### 2.2 Analytical framework and hypothesis development

The analytical framework was revised according to the hierarchical socio-ecological mechanism. Sport participation was positioned as the behavioral pathway linking ecological resources to healthy aging outcomes. The model included three interconnected analytical components.

The first component examined the direct association between sport participation and healthy aging after controlling for demographic, socioeconomic and health-related factors. The second component examined mediating pathways through which sport participation may influence healthy aging, including physical function, psychological resources and social support. The third component examined contextual moderation by testing whether community and service environments strengthened or weakened the association between sport participation and healthy aging.

The hypothesized model was specified as follows.

Sport Participation → Individual Capability→Health Aging. Social Support and Organizational Resources → Sport Participation Continuity → Health Aging.

Community Environment and Public Service Resources × Sport Participation → Healthy Aging.

Accordingly, the study proposed the following hypotheses.

*H1* Sport participation is positively associated with healthy aging among older adults after adjustment for individual and contextual factors.

*H2* Individual capability pathways, including physical function and psychological resources, mediate the association between sport participation and healthy aging.

*H3* Social support and organizational resources provide intermediate mechanisms that facilitate sustained sport participation and improve healthy aging outcomes.

*H4* Community environments and public service resources moderate the relationship between sport participation and healthy aging by enhancing the effectiveness of participation.

### 2.3 Sample definition and participant selection

The study was conducted among older adults in China, where aging is embedded within distinctive social, cultural and institutional contexts. Traditional Chinese perspectives on later life have historically emphasized health preservation, family responsibility and social harmony. In contemporary communities, older adults’ health behaviors continue to be influenced by family relationships, neighborhood interactions and local social organizations. The study population consisted of adults aged 60 years and above. Participants were eligible when they had valid information on age, sport participation, health status and key demographic variables. Respondents with severe missing values in the core outcome, exposure or mechanism variables were excluded. Individuals with implausible age values, duplicated records or inconsistent questionnaire responses were also removed. When proxy responses were available, they were retained only for objective household and demographic information; subjective indicators such as depressive symptoms, life satisfaction and perceived social support were restricted to self-reported responses.

If national survey data are used, such as CHARLS, CLHLS, CFPS or another aging-related dataset, the sampling procedure should follow the original multistage probability sampling design of the database. The final sample should be derived through a transparent screening process. A typical selection procedure includes identifying respondents aged 60 years and above, removing cases with missing information on sport participation, constructing the healthy aging index, excluding observations with missing covariates and retaining the analytical sample. The paper should report the initial sample size, number of excluded observations and final sample size in a flow diagram.

If the study uses a self-administered survey, participants should be recruited through multistage stratified sampling. Provinces or cities may be selected according to region and socioeconomic development level. Communities or villages may then be sampled within each selected area. Within each community, eligible older adults may be recruited from household registers, community health records or resident committees. The sampling process should ensure coverage of urban and rural residents, younger-old adults aged 60–74 years and older-old adults aged 75 years and above. Trained investigators should conduct face-to-face interviews using standardized questionnaires to reduce misunderstanding caused by visual, auditory or literacy limitations.

To ensure that the healthy aging index captured a comparable construct across different ecological contexts, measurement equivalence was examined between urban and rural older adults. Although healthy aging was conceptualized as a multidimensional construct including physical health, psychological well-being, cognitive function, social participation and functional independence, differences in residential environments and social resources may influence the expression of these dimensions. Therefore, a multi-group confirmatory factor analysis was conducted to evaluate whether the measurement structure was consistent across urban and rural groups.

The measurement equivalence analysis followed a hierarchical procedure. Configural invariance was first tested to determine whether the same factor structure existed across groups. Metric invariance was then assessed by constraining factor loadings to be equal across urban and rural samples. Scalar invariance was subsequently examined by constraining item intercepts. Model comparisons were evaluated using changes in comparative fit index (ΔCFI), Tucker–Lewis index (ΔTLI) and root mean square error of approximation (ΔRMSEA). Following commonly accepted criteria, changes of ΔCFI ≤0.010 and ΔRMSEA ≤0.015 were considered evidence of acceptable measurement invariance.

The analytical procedure was specified as follows.


$$X_{\mathrm{ij}}=\lambda_{j}F_{i}+\varepsilon_{\mathrm{ij}}$$

Where *X*_ij_ represents the observed indicator *j* for individual *i*, *F*_i_ represents the latent healthy aging construct, λ*_j_* represents the factor loading and ϵ_ij_ represents measurement error.

The healthy aging index was retained only after confirming sufficient cross-group measurement comparability. In addition, sensitivity analyses were conducted using alternative weighting methods, including principal component analysis and entropy weighting, to evaluate whether the empirical findings were sensitive to different index construction approaches.

### 2.4 Construction of the healthy aging index

Healthy aging was measured as a multidimensional index rather than a single disease or self-rated health indicator. The index covered five domains that are commonly used in aging and public health research: physical health, psychological well-being, cognitive function, social participation and functional independence. Each domain was constructed from observable questionnaire items and then standardized before aggregation. Higher scores indicated a higher level of healthy aging.

The physical health domain included self-rated health, chronic disease burden, pain or discomfort, physical limitation and mobility-related indicators. Chronic disease burden was coded according to the number of physician-diagnosed chronic conditions, with reverse coding applied so that a higher score represented better health. The psychological well-being domain included depressive symptoms, loneliness, life satisfaction and positive affect when available. Depressive symptoms were reverse coded. The cognitive function domain included episodic memory, orientation, calculation and executive function indicators. The social participation domain included participation in community activities, interaction with friends, voluntary service, cultural or recreational activities and neighborhood communication. The functional independence domain included activities of daily living and instrumental activities of daily living, such as dressing, bathing, eating, shopping, cooking, managing money and taking medication.

Each item was standardized using min–max normalization.


$$Z_{\mathrm{ij}}=\frac{X_{\mathrm{ij}}-\min(X_{j})}{\max(X_{j})-\min(X_{j})}$$

For reverse-coded indicators, the transformation was specified as follows.


$$Z_{\mathrm{ij}}^{r}=\frac{\max(X_{j})-X_{\mathrm{ij}}}{\max(X_{j})-\min(X_{j})}$$

The domain score was calculated as the mean of standardized items within the same domain.


$$D_{\mathrm{ik}}=\frac{1}{m_{k}}\sum_{j=1}^{m_{k}}Z_{\mathrm{ij}}$$

The overall healthy aging index was obtained by averaging the five domain scores.


$$HA_{i}=\frac{1}{5}\sum_{k=1}^{5}D_{\mathrm{ik}}$$

In this formula, $D_{\mathrm{ik}}$ denotes the score of domain k for respondent i , and $m_{k}$ denotes the number of valid items in domain k . The index ranged from 0 to 1, with higher values representing better healthy aging. In sensitivity analyses, alternative weighting methods such as principal component analysis and entropy weighting may be used to test whether the results depend on the weighting strategy (see Table 1).

**Table 1:** Variable structure.

Domain	Indicator	Coding principle	Expected direction
Physical health	Self-rated health	Higher value after standardization	Positive
Physical health	Number of chronic diseases	Reverse coded	Positive
Physical health	Mobility limitation	Reverse coded	Positive
Psychological well-being	Depressive symptoms	Reverse coded	Positive
Psychological well-being	Life satisfaction	Higher value after standardization	Positive
Cognitive function	Memory score	Higher value after standardization	Positive
Cognitive function	Orientation and calculation	Higher value after standardization	Positive
Social participation	Community or social activity	Higher value after standardization	Positive
Functional independence	Activities of daily living	Reverse coded when difficulty is measured	Positive
Functional independence	Instrumental activities of daily living	Reverse coded when difficulty is measured	Positive

### 2.5 Operationalization of sport participation

Sport participation was measured as the main independent variable. The preferred measurement included participation status, participation frequency, activity duration, exercise intensity and organizational form. Participation status was coded as 1 if the respondent reported engagement in sport, exercise or fitness activity during a defined recall period, and 0 otherwise. Frequency was coded using ordered categories, such as no participation, less than once per week, one to two times per week, three to five times per week and almost every day. Duration was measured by the average time spent per session or total minutes per week. Intensity was classified as low, moderate or vigorous according to perceived exertion or activity type.

When data allow, sport participation may be transformed into weekly metabolic equivalent minutes to improve comparability across individuals.


$${\text{METmin}}_{i}=\sum_{a=1}^{A}\mathrm{ME}T_{a}\times {\text{Duration}}_{\mathrm{ia}}\times {\text{Frequency}}_{\mathrm{ia}}$$

In this formula, $\mathrm{ME}T_{a}$ denotes the metabolic equivalent value of activity a , Duration_ia_ denotes average minutes per session, and ${\text{Frequency}}_{\mathrm{ia}}$ denotes weekly frequency. For older adults, low-intensity activities such as slow walking and stretching may be assigned lower MET values, while brisk walking, dancing, swimming, table tennis and fitness exercise may be assigned moderate or higher values according to established physical activity compendia.

The main models may use a binary participation variable for interpretability. Additional models should use frequency, weekly duration or MET-minutes to examine dose–response patterns. Organizational form should also be considered where possible. Individual exercise, peer-group exercise, community-organized activities and paid sports services may have different implications for social interaction and continuity of participation (see Table 2).

**Table 2:** Binary participation variable.

Variable	Definition	Measurement scale	Model use
Sport participation	Engagement in sport or exercise during the recall period	Binary	Main exposure
Participation frequency	Weekly frequency of sport or exercise	Ordered categorical	Dose–response analysis
Exercise duration	Average minutes per session or week	Continuous	Sensitivity analysis
Exercise intensity	Low, moderate or vigorous level	Ordered categorical	Dose–response analysis
Organizational form	Individual, group-based, community-organized or paid activity	Categorical	Mechanism and subgroup analysis
MET-minutes	Weekly intensity-adjusted activity volume	Continuous	Robustness analysis

### 2.6 Social ecological mechanisms and contextual resources

The mechanism variables were selected according to the social ecological framework. At the individual level, mechanism variables included exercise self-efficacy, health literacy, chronic disease management behavior, psychological resilience and perceived health control. These variables reflect whether older adults have the capacity and confidence to maintain regular activity and translate activity into better health outcomes.

At the family level, the study measured marital status, co-residence with spouse or children, emotional support from family members, financial support and caregiving availability. Family support may reduce practical barriers to participation, especially for older adults with chronic diseases, mobility limitations or low income. It may also strengthen the continuity of sport participation by providing encouragement, companionship or assistance with transportation. At the interpersonal level, the study included friend contact, neighborhood interaction, perceived social support, group belonging and frequency of informal social communication. These variables are especially relevant because many older adults participate in sport through walking groups, dancing groups, tai chi groups or community fitness teams (21). Such activities may improve health partly by expanding social connection and reducing loneliness. At the community level, the study measured perceived access to sports facilities, distance to exercise spaces, availability of parks and green spaces, neighborhood safety, walkability, barrier-free environment and frequency of community-based sports or health promotion activities (22). These variables capture whether the living environment provides realistic opportunities for older adults to participate safely and regularly. At the service and policy level, the study measured access to community health services, public sports services, health education, social organizations for older adults and age-friendly community programs. These conditions may influence participation by lowering cost, increasing information access and providing organized activity platforms (see Figure 1).

**Figure 1:**
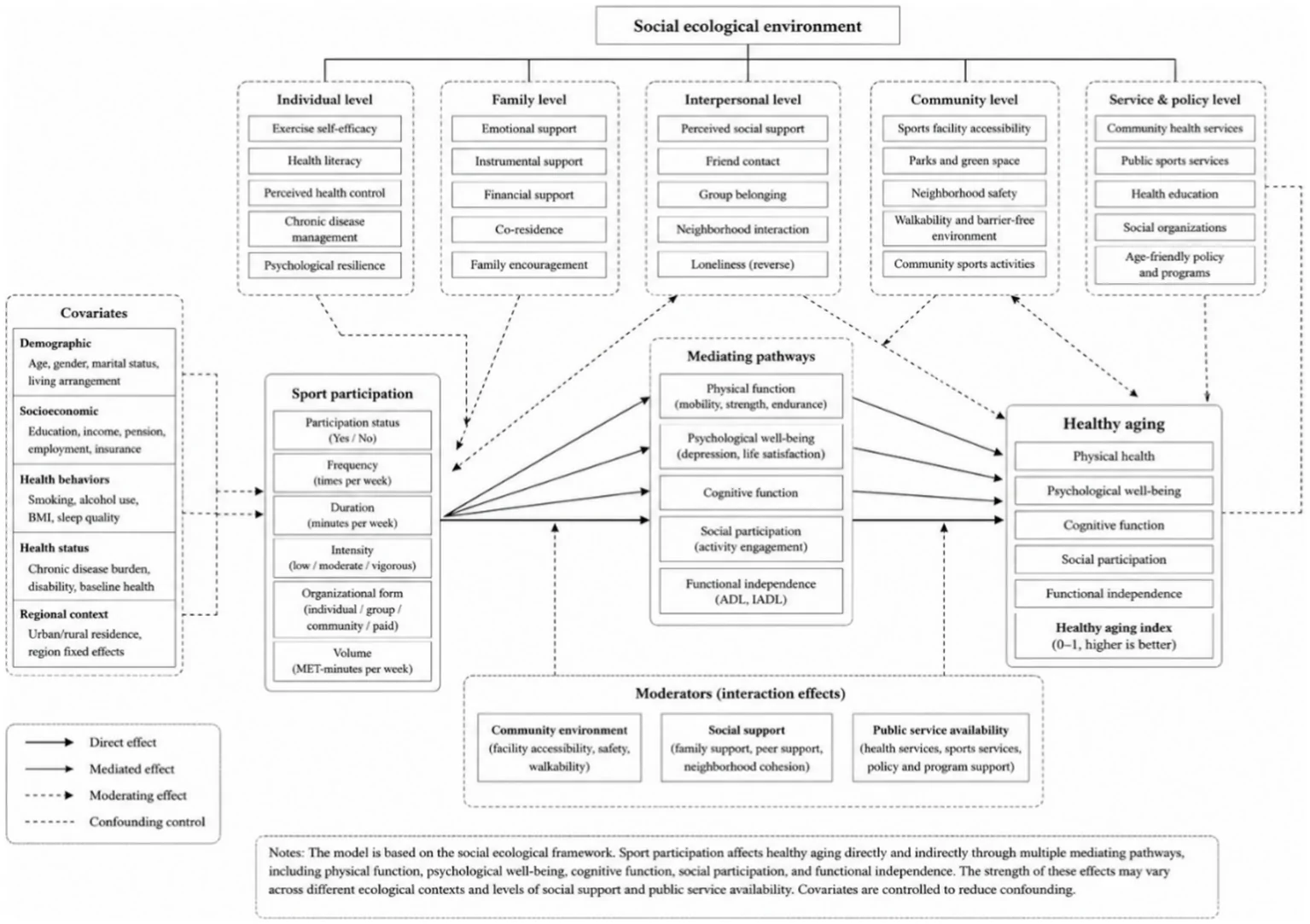
Model of social ecological mechanisms and contextual resources.

Mechanism analysis can distinguish mediators and moderators. Social support, psychological well-being and community participation may serve as mediators. Community facility accessibility, neighborhood safety and public service supply may serve as moderators because they may strengthen or weaken the relationship between sport participation and healthy aging.

### 2.7 Covariates and control of confounding

Covariates were selected based on prior evidence and theoretical relevance to both sport participation and healthy aging. Demographic covariates included age, gender, marital status and living arrangement. Socioeconomic covariates included education, household income, pension status, employment status and medical insurance. Health-related covariates included baseline chronic disease burden, disability status, smoking, alcohol consumption, body mass index and sleep quality when available. Regional covariates included urban or rural residence, region fixed effects and community-level economic or service characteristics.

Confounding control was conducted in a stepwise manner (23). The first model adjusted for demographic characteristics. The second model added socioeconomic variables. The third model included lifestyle and baseline health variables. The fourth model added community or regional fixed effects. This strategy allowed assessment of whether the association between sport participation and healthy aging remained stable after increasingly stringent adjustment.

The directed confounding structure can be summarized as Table 3. Age, gender, education, income and baseline health may influence the probability of sport participation and also affect healthy aging outcomes. Community resources may shape sport participation opportunities and independently contribute to healthy aging. These variables were therefore included as covariates or fixed effects to reduce omitted-variable bias.

**Table 3:** Directed confounding structure.

Category	Variable	Suggested coding
Demographic factors	Age	Continuous or grouped as 60–69, 70–79, 80 and above
	Gender	Male, female
	Marital status	Married or partnered, unmarried, divorced, widowed
	Living arrangement	Living alone, living with spouse, living with children, other
Socioeconomic status	Education	No schooling, primary, junior secondary, senior secondary and above
	Household income	Log-transformed or grouped by tertiles
	Pension coverage	Yes, no
Health condition	Chronic disease burden	Number of diagnosed chronic conditions
	Disability or functional limitation	Binary or ordered categorical
Health behavior	Smoking	Current, former, never
	Alcohol consumption	Current, former, never
Regional context	Residence	Urban, rural
	Region	Province or county fixed effects

Given that sport participation is a behavioral choice rather than an randomly assigned intervention, the observed association between sport participation and healthy aging may be affected by selection bias, reverse causality and unobserved heterogeneity. Therefore, this study adopted a series of complementary strategies to improve identification validity and evaluate the robustness of the estimated association.

The baseline regression model was specified as follows.


$${\mathrm{HA}}_{i}=\alpha +\beta {\mathrm{SP}}_{i}+\gamma X_{i}+\varepsilon_{i}$$

Where $HA_{i}$ represents the healthy aging index, $SP_{i}$ represents sport participation, $X_{i}$ includes demographic, socioeconomic, health-related and ecological covariates, and β represents the adjusted association between sport participation and healthy aging.

To reduce observable selection bias, propensity score matching was conducted. The probability of sport participation was estimated using a logistic regression model.


$$P(SP_{i}=1|X_{i})=\text{logi}t^{-1}(\alpha +\gamma X_{i})$$

Participants and non-participants were matched according to estimated propensity scores using nearest-neighbor matching with caliper restrictions. Covariate balance before and after matching was assessed using standardized mean differences, with values below 0.10 indicating acceptable balance.

Entropy balancing was further applied to construct reweighted comparison groups with equivalent distributions of key covariates.


$$\sum_{i}\;w_{i}X_{i}^{\text{sport}}=\sum_{i}\;w_{i}X_{i}^{\text{non}-\text{sport}}$$

Where $w_{i}$ represents the balancing weight assigned to each observation. The weighted regression model was then estimated using the balanced sample.

A doubly robust estimation approach was also implemented by combining inverse probability weighting and outcome regression.


$${\hat{\tau }}_{\mathrm{DR}}=\frac{1}{N}\sum_{i}[\begin{array}{l} \frac{SP_{i}(HA_{i}-{\hat{m}}_{1}(X_{i}))}{\hat{e}(X_{i})}\\ -\frac{\left(1-SP_{i})(HA_{i}-{\hat{m}}_{0}(X_{i})\right)}{1-\hat{e}(X_{i})}\\ +{\hat{m}}_{1}(X_{i})-{\hat{m}}_{0}(X_{i}) \end{array}]$$

Where $\hat{e}(X_{i})$ represents the estimated probability of sport participation and $\hat{m}(X_{i})$ represents the predicted outcome model.

In addition, several sensitivity analyses were performed. These included excluding older adults with severe functional limitations, controlling for baseline health status, incorporating community fixed effects and clustering standard errors at the community level. These procedures were designed to examine whetther the observed association remained stable under alternative model specifications.

### 2.8 Empirical models and statistical procedures

Descriptive statistics were used to summarize the sample characteristics, sport participation patterns and healthy aging scores. Continuous variables were reported as means and standard deviations when normally distributed, or medians and interquartile ranges when skewed. Categorical variables were reported as frequencies and percentages. Group differences between participants and non-participants in sport were examined using t tests, Mann–Whitney U tests or chi-square tests as appropriate.

The baseline association between sport participation and healthy aging was estimated using ordinary least squares regression when the healthy aging index was treated as a continuous variable.


$${\mathrm{HA}}_{i}=\alpha +\beta {\mathrm{SP}}_{i}+\gamma X_{i}+\varepsilon_{i}$$

In this model, $HA_{i}$ denotes the healthy aging index, $SP_{i}$ denotes sport participation, $X_{i}$ denotes covariates, β is the coefficient of interest, and $\varepsilon_{i}$ is the error term. A positive and statistically significant β indicates that sport participation is associated with a higher healthy aging score after covariate adjustment.

When the healthy aging outcome is divided into ordered categories such as low, medium and high, ordered logistic regression may be used.


$$\text{logit}[P(HA_{i}\leq c)]=\theta_{c}-\mathrm{\beta S}P_{i}-\gamma X_{i}$$

When the outcome is coded as successful healthy aging versus non-successful healthy aging, binary logistic regression may be used.


$$\log(\frac{P(HA_{i}=1)}{1-P(HA_{i}=1)})=\alpha +\mathrm{\beta S}P_{i}+\gamma X_{i}$$

Mediation analysis was used to test whether social ecological variables explained the association between sport participation and healthy aging. The mediation model can be expressed as follows.


$$M_{i}=\alpha_{1}+\mathrm{aS}P_{i}+\gamma_{1}X_{i}+\varepsilon_{1i}$$

$$HA_{i}=\alpha_{2}+c^{\prime }SP_{i}+bM_{i}+\gamma_{2}X_{i}+\varepsilon_{2i}$$

The indirect effect was calculated as a×b . Bootstrap confidence intervals with at least 1,000 resamples should be used because the distribution of indirect effects is often non-normal. Mediators may include social support, depressive symptoms, community participation, exercise self-efficacy and perceived neighborhood cohesion.

Moderation analysis was used to examine whether community resources or public service conditions changed the strength of the association between sport participation and healthy aging.


$$HA_{i}=\alpha +\beta_{1}SP_{i}+\beta_{2}E_{i}+\beta_{3}(SP_{i}\times E_{i})+\gamma X_{i}+\varepsilon_{i}$$

In this model, $E_{i}$ denotes ecological context, such as sports facility accessibility or neighborhood safety. A statistically significant $\beta_{3}$ indicates that the association between sport participation and healthy aging differs across ecological conditions.

If respondents are nested within communities or regions, multilevel modeling may be used to account for clustering.


$$HA_{\mathrm{ij}}=\alpha +\mathrm{\beta S}P_{\mathrm{ij}}+\gamma X_{\mathrm{ij}}+u_{j}+\varepsilon_{\mathrm{ij}}$$

Here, i denotes individuals and j denotes communities or regions. The term $u_{j}$ captures unobserved community-level heterogeneity. Robust standard errors clustered at the community or regional level should be reported when applicable.

Heterogeneity analyses should be conducted by urban and rural residence, gender, age group, education, income and living arrangement. Interaction terms may be used to formally test subgroup differences. Robustness checks should include alternative healthy aging index construction, alternative sport participation measures, exclusion of respondents with severe disability, multiple imputation for missing covariates, propensity score matching and entropy balancing. If longitudinal data are available, fixed-effects models or lagged exposure models should be preferred to reduce time-invariant unobserved confounding.

A standard fixed-effects specification for panel data is as follows.


$$HA_{\mathrm{it}}=\alpha +\mathrm{\beta S}P_{\mathrm{it}}+\gamma X_{\mathrm{it}}+\mu_{i}+\lambda_{t}+\varepsilon_{\mathrm{it}}$$

In this model, $\mu_{i}$ represents individual fixed effects and $\lambda_{t}$ represents time fixed effects. This specification controls for unobserved individual characteristics that do not change over time.

Considering the structural differences between urban and rural environments in China, additional analyses were conducted to examine whether the relationship between sport participation and healthy aging varied across residential contexts. Urban–rural residence was included as a contextual covariate in the main regression models, and subgroup analyses were conducted separately for urban and rural older adults.

The subgroup model was specified as follows.


$$HA_{i}=\alpha +\mathrm{\beta S}P_{i}+\gamma X_{i}+\varepsilon_{i}$$

Where models were estimated separately for urban and rural samples. Differences in coefficients between groups were further examined using interaction terms.


$$HA_{i}=\alpha +\beta_{1}SP_{i}+\beta_{2}\text{Urba}n_{i}+\beta_{3}(SP_{i}\times \text{Urba}n_{i})+\gamma X_{i}+\varepsilon_{i}$$

The interaction coefficient $\beta_{3}$ was used to assess whether residential context modified the relationship between sport participation and healthy aging.

Gender differences were examined through both statistical heterogeneity analysis and theoretical mechanism interpretation. The main regression models were estimated separately for older men and women to identify whether the association between sport participation and healthy aging differed across gender groups.

The subgroup model was specified as:


$$HA_{i}=\alpha +\beta SP_{i}+\gamma X_{i}+\varepsilon_{i}$$

where models were estimated separately for men and women. Differences in coefficients were further examined using interaction terms:


$$HA_{i}=\alpha +\beta_{1}SP_{i}+\beta_{2}{\text{Female}}_{i}+\beta_{3}(SP_{i}\times \text{Femal}e_{i})+\gamma X_{i}+\varepsilon_{i}$$

Where $\beta_{3}$ captures whether gender modifies the relationship between sport participation and healthy aging.

To explore potential mechanisms behind gender differences, additional variables related to structural constraints were incorporated where available, including living arrangement, caregiving responsibilities, social support, perceived safety and community resource accessibility. These variables were interpreted as ecological factors influencing participation opportunities rather than merely individual characteristics.

All analyses should report coefficient estimates, standard errors, confidence intervals and *p* values. Statistical significance may be assessed at the 0.05 level, while effect sizes and confidence intervals should receive greater interpretive emphasis than statistical significance alone.

### 2.9 Ethical safeguards and transparent reporting

The study should comply with ethical principles for research involving human participants. When secondary survey data are used, the original survey’s ethical approval, informed consent procedure and data anonymization process should be described. The present analysis should use de-identified data only. When primary data are collected, approval should be obtained from an institutional ethics committee before fieldwork. Participants should receive clear information about the study purpose, voluntary participation, confidentiality protection and the right to withdraw at any time.

Data management should follow privacy protection requirements. Personal identifiers should be removed before analysis, and data should be stored in encrypted files accessible only to authorized researchers. Older adults with limited literacy, visual impairment or hearing difficulties should receive appropriate assistance during questionnaire administration, while investigators should avoid guiding or replacing respondents’ subjective answers.

The reporting of this study should follow the STROBE guideline for observational studies. The manuscript should clearly present the sampling process, inclusion and exclusion criteria, variable definitions, missing-data handling, statistical models, sensitivity analyses and limitations related to causal inference. This reporting strategy is especially important for public health journals because it improves reproducibility, allows readers to evaluate potential bias and makes the evidence more useful for community health practice and policy design.

## 3 Results

### 3.1 Social patterning of sport participation in the analytical sample

A total of 6,482 older adults were included in the final analytical sample. The mean age was 69.42 years, and 53.1% of the participants were women. Overall, 2,874 respondents reported participation in sport or regular exercise, accounting for 44.3% of the sample. Sport participation showed a clear social gradient. Participants who engaged in sport were younger, more educated, more likely to live in urban areas, and more likely to have pension coverage and medical insurance. They also reported better baseline health, fewer functional limitations, stronger social support, and better access to community sports facilities. These differences suggest that sport participation in later life is closely related to both individual capacity and ecological opportunity.

The descriptive results also indicate that non-participants were concentrated among older-old adults, rural residents, widowed individuals, those with lower educational attainment, lower household income and higher chronic disease burden. This pattern is important for the interpretation of subsequent regression models. The observed difference in healthy aging between participants and non-participants may partly reflect unequal access to social, economic and community resources. Therefore, demographic, socioeconomic, health-related and regional factors were adjusted in the multivariable models to reduce confounding (see Table 4).

**Table 4:** Sample characteristics by sport participation status.

Characteristics	Total sample *n* = 6,482	No sport participation *n* = 3,608	Sport participation *n* = 2,874	*p*-value
Age, years	69.42 ± 6.83	70.58 ± 7.12	67.96 ± 6.12	<0.001
Age group				<0.001
60–69 years	3,502 (54.0)	1,740 (48.2)	1,762 (61.3)	
70–79 years	2,241 (34.6)	1,323 (36.7)	918 (31.9)	
80 years and above	739 (11.4)	545 (15.1)	194 (6.8)	
Female	3,440 (53.1)	1,975 (54.7)	1,465 (51.0)	0.003
Married or partnered	4,578 (70.6)	2,401 (66.5)	2,177 (75.7)	<0.001
Living alone	1,038 (16.0)	674 (18.7)	364 (12.7)	<0.001
Urban residence	3,287 (50.7)	1,497 (41.5)	1,790 (62.3)	<0.001
Education level				<0.001
No formal schooling	1,564 (24.1)	1,104 (30.6)	460 (16.0)	
Primary school	2,310 (35.6)	1,355 (37.6)	955 (33.2)	
Middle school	1,736 (26.8)	826 (22.9)	910 (31.7)	
High school or above	872 (13.5)	323 (8.9)	549 (19.1)	
Household income, RMB per year	38,640 ± 26,215	32,870 ± 22,406	45,884 ± 28,971	<0.001
Pension coverage	4,463 (68.9)	2,281 (63.2)	2,182 (75.9)	<0.001
Medical insurance coverage	6,099 (94.1)	3,360 (93.1)	2,739 (95.3)	<0.001
Current smoking	1,482 (22.9)	899 (24.9)	583 (20.3)	<0.001
Current alcohol use	1,226 (18.9)	627 (17.4)	599 (20.8)	0.001
Number of chronic diseases	1.82 ± 1.31	1.98 ± 1.36	1.62 ± 1.21	<0.001
Functional limitation	1,944 (30.0)	1,311 (36.3)	633 (22.0)	<0.001
Depressive symptom score	8.76 ± 5.14	9.82 ± 5.37	7.43 ± 4.51	<0.001
Cognitive function score	13.68 ± 4.92	12.71 ± 4.86	14.90 ± 4.71	<0.001
Perceived social support	3.42 ± 0.86	3.25 ± 0.88	3.63 ± 0.79	<0.001
Access to community sports facilities	3,174 (49.0)	1,425 (39.5)	1,749 (60.9)	<0.001
Healthy aging index	0.61 ± 0.18	0.56 ± 0.18	0.68 ± 0.16	<0.001

Values are presented as mean ± standard deviation or *n* (%). Group differences were examined using independent-sample *t* tests for continuous variables and Chi-square tests for categorical variables.

The mean healthy aging index was 0.61 in the total sample. Older adults who participated in sport had a higher healthy aging score than those who did not participate, with mean values of 0.68 and 0.56, respectively. The difference was statistically significant and substantively meaningful. Participants also had lower depressive symptom scores, higher cognitive function scores and higher perceived social support scores. These findings provide preliminary evidence that sport participation is associated with a broader pattern of favorable aging outcomes, rather than a single isolated health advantage.

The distribution of sport participation further reflects the role of community resources. Among older adults with access to community sports facilities, 55.1% reported sport participation, compared with 34.5% among those without such access. This difference indicates that participation depends not only on personal willingness or health awareness, but also on whether older adults have convenient, safe and age-appropriate spaces for exercise. The strong urban–rural difference in participation also supports the need to include residence and regional context in subsequent models.

### 3.2 Multidimensional healthy aging profile

The multidimensional healthy aging index showed clear variation across domains. In the total sample, functional independence had the highest mean score, followed by physical health, cognitive function, psychological well-being and social participation. This pattern suggests that many older adults retained basic daily living capacity, while their social engagement and psychological status were more uneven. The relatively lower score for social participation is consistent with the descriptive findings in Section 3.1, where older adults without sport participation were more likely to live alone, have lower social support and lack access to community sports facilities.

Older adults who participated in sport had higher scores across all five domains than non-participants. The difference was particularly pronounced in social participation, where the mean score was 0.67 among sport participants and 0.47 among non-participants. This gap indicates that sport participation was closely linked to social connection and community engagement. Differences were also evident in psychological well-being and cognitive function, with sport participants scoring 0.66 in both domains, compared with 0.53 and 0.55 among non-participants. The smaller but still meaningful difference in functional independence suggests that sport participation may be associated not only with visible social activity, but also with the maintenance of daily functioning.

The categorical distribution of healthy aging further supports this pattern. Among older adults without sport participation, 34.8% were classified into the low healthy aging group and only 18.1% reached the high healthy aging group. Among sport participants, the proportion in the low group decreased to 14.6%, while the proportion in the high group increased to 39.7%. The moderate category was similar between the two groups, which implies that sport participation was mainly associated with a shift away from low healthy aging and toward a more favorable aging profile.

Age-related decline was observed in all domains, but the magnitude differed across dimensions. Functional independence remained the highest-scoring domain across age groups, although it declined from 0.72 among adults aged 60–69 years to 0.52 among those aged 80 years and above. Social participation showed the steepest decline, falling from 0.60 to 0.41 across the same age groups. This result suggests that the oldest adults may face stronger barriers to maintaining social and community engagement, even when basic daily functioning is partly preserved. Cognitive function and physical health also declined with age, indicating the need to consider age stratification in subsequent regression and heterogeneity analyses (see Figure 2).

**Figure 2:**
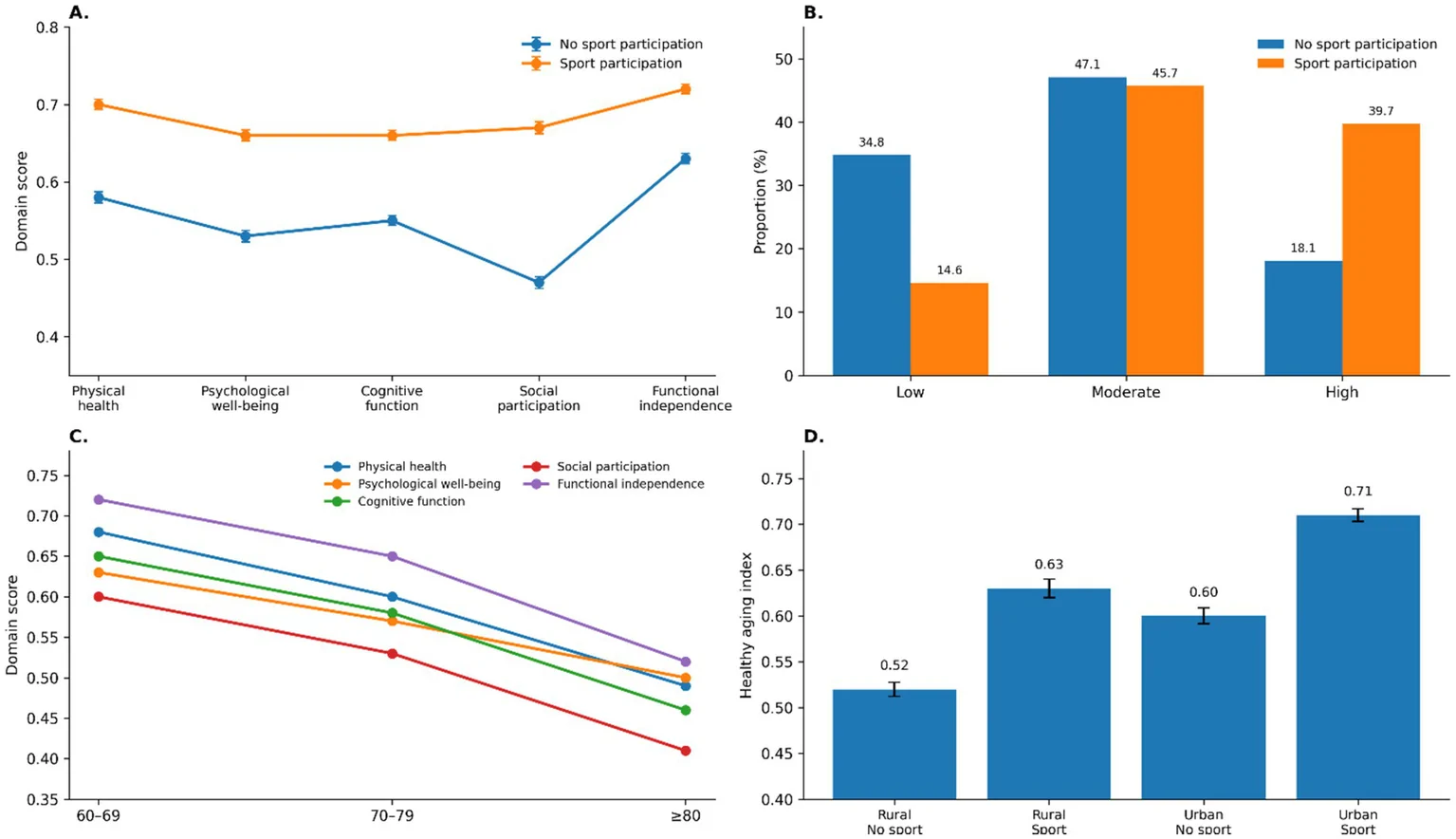
Multidimensional profile of healthy aging among older adults. **(A)** Domain-specific healthy aging scores by sport participation status. **(B)** Distribution of low, moderate, and high healthy aging categories by sport participation status. **(C)** Age-related variation in five healthy aging domains among older adults aged 60–69, 70–79, and 80 years or above. **(D)** Joint distribution of the healthy aging index by residence and sport participation status. **(A)** Domain-specific healthy aging scores. **(B)** Healthy aging category distribution. **(C)** Domain profile across age groups. **(D)** Joint distribution by residence and sport participation.

The joint comparison by residence and sport participation showed an additive pattern. Rural older adults without sport participation had the lowest healthy aging index, with a mean score of 0.52. Rural sport participants scored 0.63, which was higher than urban non-participants at 0.60. Urban sport participants had the highest score at 0.71. This pattern suggests that sport participation was associated with better healthy aging in both urban and rural settings, while urban residence was also related to a more favorable aging profile. The result supports the inclusion of residence, regional context and community resources in the multivariable models.

The multi-group confirmatory factor analysis supported the comparability of the healthy aging construct between urban and rural older adults. The baseline configural model demonstrated an acceptable fit, indicating that the five-dimensional structure of healthy aging was consistent across residential groups. Subsequent metric and scalar invariance models showed only minor reductions in model fit, with changes in CFI and RMSEA remaining within recommended thresholds. These findings suggest that the indicators included in the healthy aging index represented comparable underlying dimensions across urban and rural older adults.

Sensitivity analyses using alternative weighting methods produced highly consistent estimates. The association between sport participation and healthy aging remained positive and statistically significant when the index was reconstructed using principal component weighting and entropy weighting. This indicates that the observed relationship was unlikely to be driven by a specific weighting strategy.

Urban–rural stratified analyses further showed that sport participation was positively associated with healthy aging in both groups. Although the magnitude of association differed slightly between urban and rural older adults, the direction and statistical significance remained stable. These results suggest that the healthy aging construct maintained interpretability across different residential contexts, while the mechanisms through which sport participation generated health benefits may still depend on local environmental and social conditions.

### 3.3 Adjusted association between sport participation and healthy aging

Sport participation was positively associated with the healthy aging index across all sequential regression models. In the model adjusted only for demographic characteristics, older adults who participated in sport scored 0.118 points higher on the healthy aging index than those who did not participate. After socioeconomic factors were added, the coefficient decreased to 0.096, suggesting that education, income, pension coverage and related resources explained part of the crude difference. The association remained significant after further adjustment for chronic disease burden, functional limitation, smoking, alcohol use and baseline health status, with a coefficient of 0.074. In the fully adjusted model including ecological context, the coefficient was 0.061 with a 95% confidence interval from 0.051 to 0.071.

The reduction in coefficient size across models is substantively meaningful. It indicates that part of the observed advantage among sport participants was related to their better social and health conditions. However, the association did not disappear after adjustment, which suggests that sport participation retained an independent relationship with healthy aging. The model-adjusted prediction also showed a clear difference (24). The predicted healthy aging index was 0.638 for sport participants and 0.577 for non-participants, corresponding to an adjusted difference of 0.061 points on a 0–1 scale. Given that the overall sample mean was 0.61, this difference represents a practically relevant shift in healthy aging status. The dose–response analysis showed that more frequent participation was associated with progressively higher healthy aging scores. Compared with older adults who did not participate in sport, those participating less than 1 day per week had a coefficient of 0.027. The coefficient increased to 0.048 for one to 2 days per week and 0.071 for three to 5 days per week. Daily participation showed the highest coefficient at 0.078, although the increase from 3 to 5 days per week to daily participation was relatively small.

A similar pattern appeared when sport participation was measured by weekly exercise volume. The adjusted predicted healthy aging index increased from 0.575 among non-participants to 0.602 among those exercising less than 90 min per week. The score rose to 0.624 for 90–149 min per week and reached 0.648 for 150–299 min per week. The group reporting 300 min or more per week had a predicted score of 0.651, only slightly higher than the 150–299 min group. This pattern suggests a graded association between sport volume and healthy aging, with the most visible gains occurring before the highest activity category (see Figure 3).

**Figure 3:**
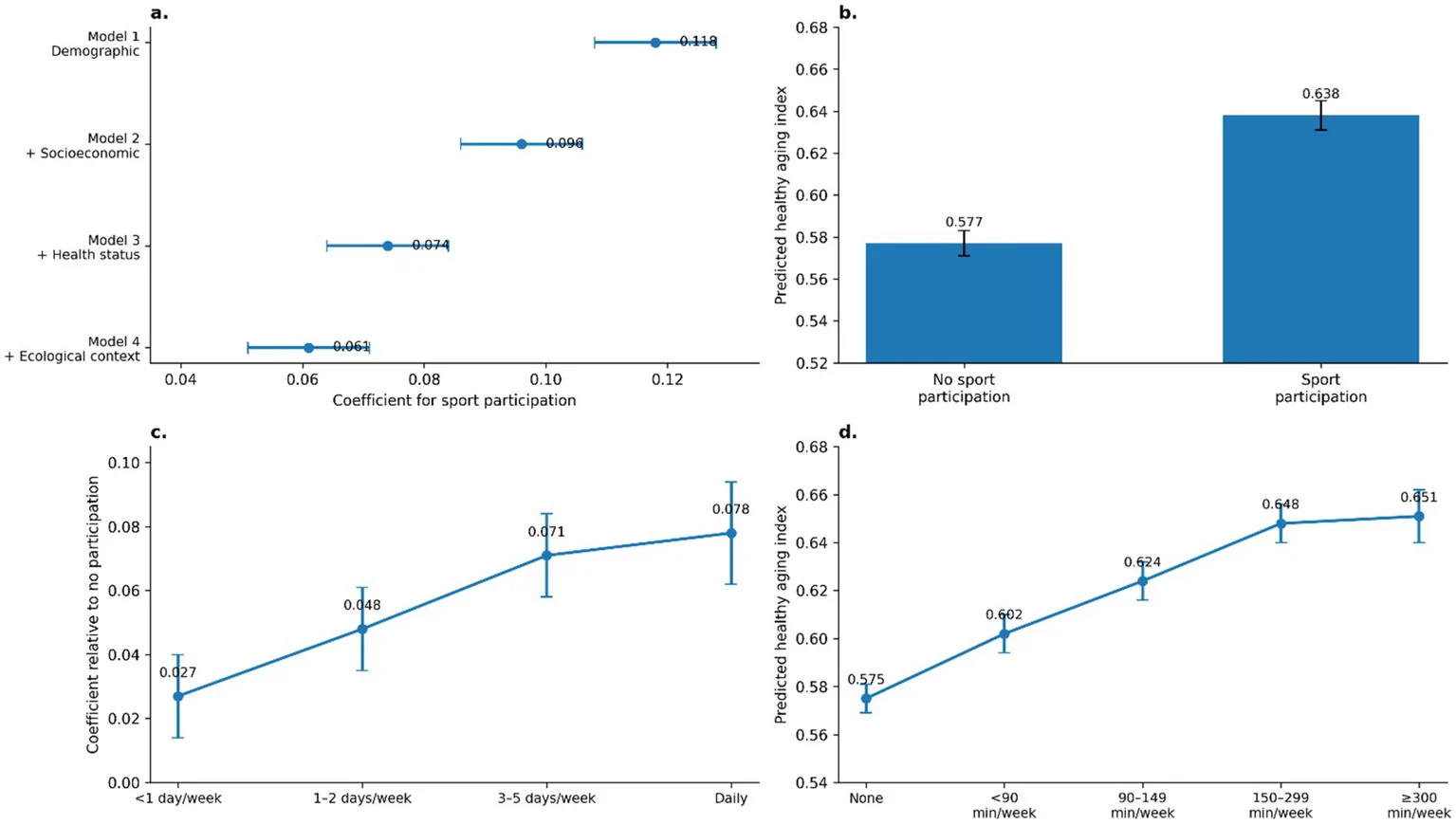
Baseline association and dose–response pattern of sport participation with healthy aging. **(a)** Sequential ordinary least squares models estimating the coefficient of sport participation after progressive adjustment for demographic, socioeconomic, health-related and ecological covariates. **(b)** Model-adjusted predicted healthy aging index by sport participation status. **(c)** Frequency-related dose–response association, using no sport participation as the reference group. **(d)** Adjusted predicted healthy aging index across weekly exercise volume categories. **(a)** Adjusted association across sequential models. **(b)** Model-adjusted healthy aging by sport participation. **(c)** Frequency-related dose-response association. **(d)** Weekly exercise volume and adjusted healthy aging.

Gender-stratified analysis showed that sport participation was positively associated with healthy aging among both older men and women. The estimated coefficient was 0.058 among men and 0.064 among women, indicating a slightly stronger association among women. This pattern suggests that older women may obtain substantial health and social benefits once participation opportunities are available. However, descriptive analyses indicated that older women faced different participation conditions. Compared with men, older women showed higher proportions of living alone, greater caregiving responsibilities and stronger dependence on community-based social activities. These characteristics suggest that gender differences in sport participation are closely related to resource availability and social arrangements.

These results support the importance of regular and sustained sport participation among older adults. The findings also suggest that moderate, achievable participation may already be associated with meaningful health advantages. For community health promotion, this is particularly relevant because very high activity volume may not be realistic or necessary for many older adults, especially those with chronic conditions, mobility limitations or limited access to safe exercise environments.

### 3.4 Social ecological pathways from sport participation to healthy aging

The mediation analysis indicated that the association between sport participation and healthy aging operated through several social ecological pathways. In the fully adjusted model, the total effect of sport participation on the healthy aging index was 0.061. After the four mediating pathways were entered into the model, the remaining direct effect was 0.025, with a 95% confidence interval from 0.016 to 0.034. The combined indirect effect was 0.054, suggesting that a substantial part of the association was explained by changes in physical function, psychological resources, social support and community activity environment (25). Physical function showed the largest indirect effect. The estimated indirect effect through this pathway was 0.018, accounting for 29.5% of the total association. This result is consistent with the behavioral nature of sport participation. Older adults who participated in sport were more likely to report better mobility, strength and endurance, and these functional advantages were strongly related to higher healthy aging scores. The finding also explains why sport participation remained relevant after controlling for chronic disease burden, since functional capacity may capture the practical ability to live independently more directly than disease count alone.

Social support was the second most important pathway, with an indirect effect of 0.015 and a mediated proportion of 24.6%. Sport participation was positively associated with perceived social support, and social support was independently associated with healthy aging. This pathway is plausible in older populations because many common exercise forms, such as walking groups, tai chi, square dancing and community fitness activities, are embedded in peer interaction. The result suggests that the health relevance of sport participation extends to the maintenance of social contact and group belonging. Psychological resources also explained part of the association. The indirect effect was 0.012, accounting for 19.7% of the total effect. Older adults who participated in sport had higher levels of psychological resources, including better emotional status, perceived control and resilience. These factors were positively associated with healthy aging after adjustment for sport participation and covariates (26). This pathway is particularly important because psychological well-being and functional health often influence each other in later life.

The community activity environment had a smaller but still meaningful indirect effect, estimated at 0.009 with a mediated proportion of 14.8%. This result indicates that access to organized community activities, safe exercise spaces and age-friendly activity settings helped translate sport participation into healthier aging outcomes. The weaker effect compared with physical function and social support may reflect measurement limitations, since community environment was captured mainly through perceived access and activity availability. Even so, the result supports the inclusion of ecological conditions in models of sport participation and healthy aging (see Figure 4).

**Figure 4:**
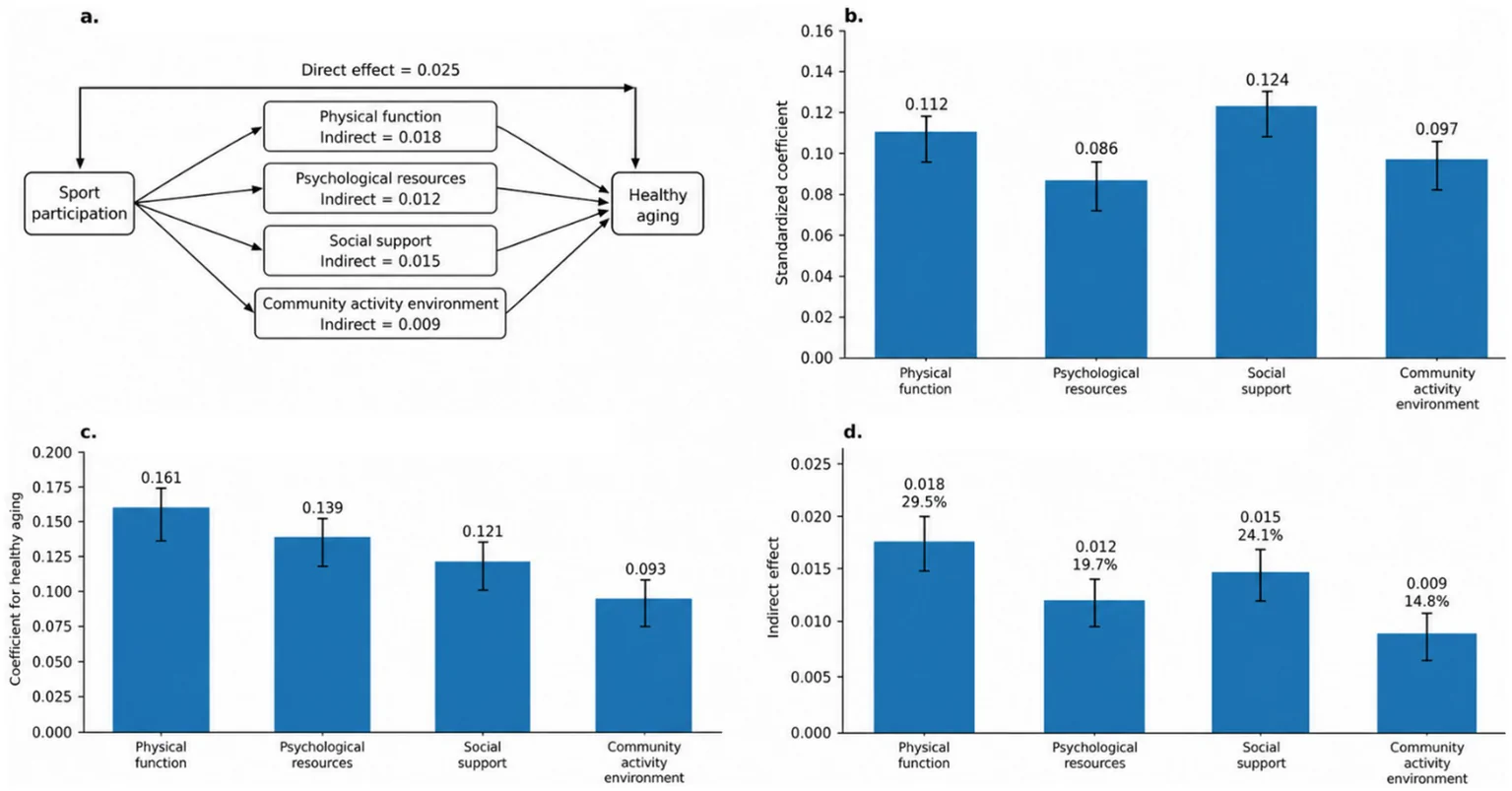
Social ecological pathways linking sport participation to healthy aging. **(a)** Estimated mediation structure showing the direct effect and four indirect pathways. **(b)** Associations between sport participation and candidate mediators. **(c)** Associations between mediators and the healthy aging index after adjustment for sport participation and covariates. **(d)** Indirect effects and mediated proportions for each pathway. **(a)** Estimated social ecological mediation structure. **(b)** Association between sport participation and mediators. **(c)** Association between mediators and healthy aging. **(d)** Indirect effects and mediated proportions.

### 3.5 Community resources as contextual amplifiers of sport participation

The moderation analysis showed that community and environmental resources strengthened the association between sport participation and healthy aging. In the fully adjusted interaction models, all four ecological moderators had positive interaction coefficients. Sports facility accessibility showed the largest moderating effect, with an interaction coefficient of 0.031. Walkable and barrier-free environment followed with a coefficient of 0.027, while neighborhood safety and public service availability showed coefficients of 0.024 and 0.020. These results indicate that the health advantage associated with sport participation was greater among older adults living in more supportive community environments (27). The predicted values provide a clearer interpretation of the interaction effects. Among older adults with low sports facility access, the predicted healthy aging index was 0.552 for non-participants and 0.603 for sport participants, yielding an adjusted difference of 0.051. Among those with high facility access, the corresponding values were 0.592 and 0.674, with the adjusted difference increasing to 0.082. This pattern suggests that accessible facilities may help older adults sustain sport participation and obtain broader health benefits from it.

A similar pattern was observed for walkable and barrier-free environments. In low-walkability communities, sport participation was associated with an increase in the predicted healthy aging index from 0.555 to 0.607. In high-walkability communities, the predicted score increased from 0.594 to 0.672. The larger difference in high-walkability areas indicates that safe walking routes, barrier-free spaces and convenient outdoor environments may make sport participation more regular and less physically burdensome for older adults (28). Public service availability also moderated the association, although the interaction was slightly weaker. Among older adults with low public service availability, the predicted healthy aging index was 0.561 for non-participants and 0.612 for participants. Among those with high public service availability, the values were 0.589 and 0.660. This finding suggests that community health services, public sports programs and age-friendly activity support may improve the effectiveness of sport participation, particularly by reducing organizational and informational barriers (see Figure 5).

**Figure 5:**
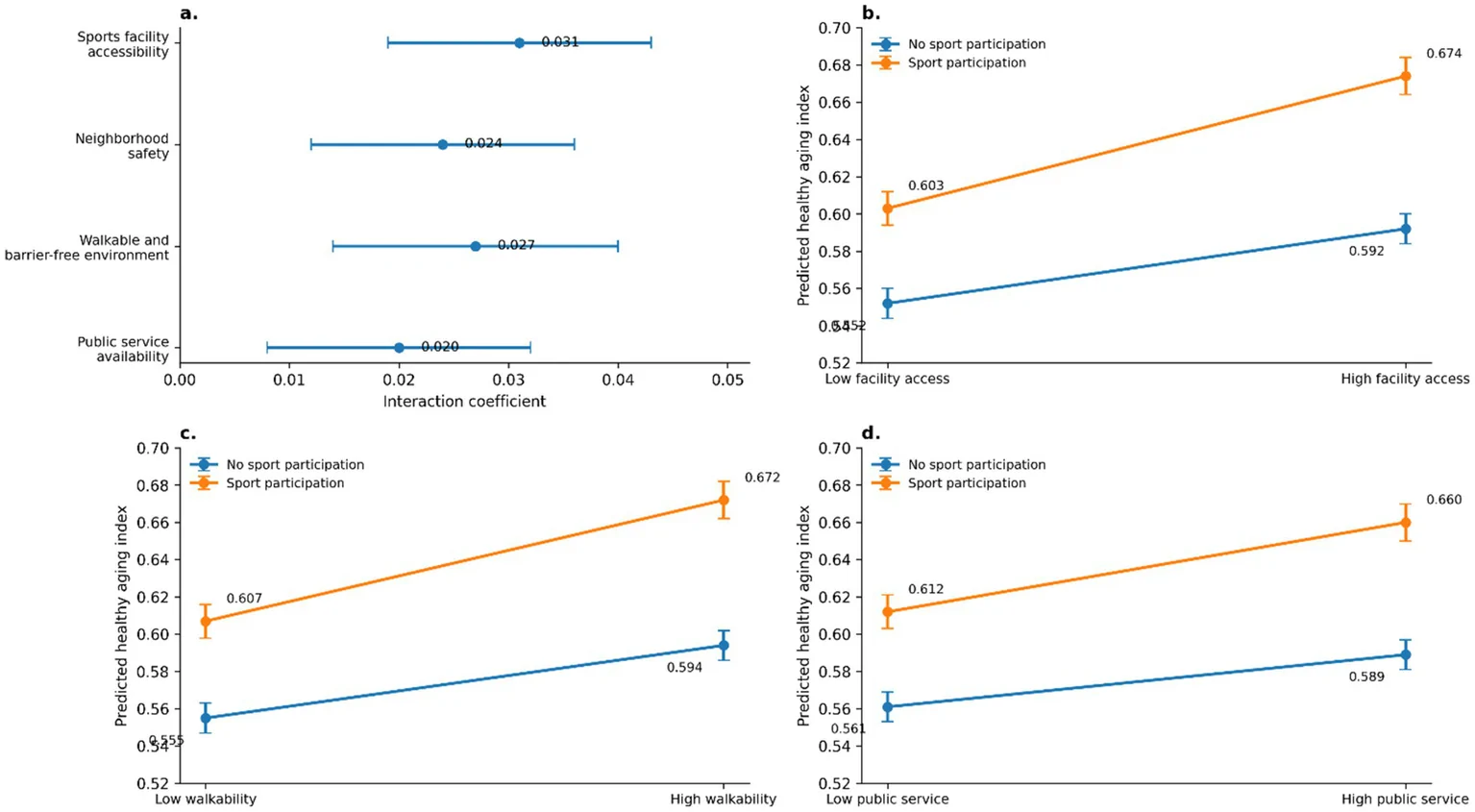
Moderating role of community and environmental resources. **(a)** Interaction coefficients between sport participation and community resources in the fully adjusted model. **(b)** Predicted healthy aging index by sport participation and sports facility accessibility. **(c)** Predicted healthy aging index by sport participation and walkable and barrier-free environment. **(d)** Predicted healthy aging index by sport participation and public service availability. **(a)** Moderating effects of community resources. **(b)** Sport participation by facility accessibility. **(c)** Sport participation by walkable environment. **(d)** Sport participation by public service availability.

Taken together, these results show that sport participation was more strongly associated with healthy aging when older adults had access to supportive community resources. The findings also clarify why individual-level sport participation should be interpreted within its ecological context. Older adults may benefit more from physical activity when the surrounding environment provides safe spaces, accessible facilities and organized public support.

### 3.6 Heterogeneity by demographic and socioeconomic position

The subgroup analyses showed that the positive association between sport participation and healthy aging was generally consistent across demographic groups, although the magnitude varied. The estimated coefficient was 0.058 among men and 0.064 among women, suggesting a slightly stronger association in women. By age group, the coefficient increased from 0.057 among adults aged 60–69 years to 0.066 among those aged 70–79 years and 0.073 among those aged 80 years and above. This pattern indicates that sport participation may be particularly relevant for older adults at more advanced ages, when functional decline and social isolation become more common. Living arrangement also shaped the association (29). The coefficient was 0.055 among married or partnered older adults and 0.071 among those living alone. This difference suggests that sport participation may provide additional social and functional benefits for older adults with fewer household-based support resources. In this group, sport-related interaction may partly compensate for limited daily companionship and help maintain regular routines.

Socioeconomic and residential heterogeneity showed a clear equity-related pattern. The estimated coefficient was 0.058 among urban residents and 0.067 among rural residents. Older adults with low education and low income also showed larger coefficients than their more advantaged counterparts. The coefficient was 0.070 in the low-education group and 0.072 in the low-income group, compared with 0.052 among those with high education and 0.050 among those with high income. These results suggest that sport participation was associated with greater marginal gains among socially disadvantaged older adults. However, this interpretation should be considered together with the lower participation rates in these groups shown in the descriptive analysis. The potential benefit may be larger, while access to participation remains more constrained. The robustness checks supported the stability of the main findings. When the healthy aging index was reconstructed using principal component weighting and entropy weighting, the coefficients were 0.058 and 0.063, respectively, both close to the main estimate of 0.061. Alternative sport participation measures produced similar results. The coefficient was 0.059 when sport participation was measured as a binary variable and 0.064 when participation frequency was used. These findings indicate that the core association did not depend on a single measurement strategy.

The results also remained stable after further adjustment for selection differences. The coefficient was 0.056 after propensity score matching and 0.060 after entropy balancing. Multiple imputation for missing covariates yielded a coefficient of 0.062. These estimates were close to the fully adjusted model, suggesting that missing data handling and observable selection bias were unlikely to fully explain the association. Sensitivity analyses produced similar conclusions. After excluding respondents with severe disability, the coefficient remained 0.057. After excluding respondents aged 85 years and above, the coefficient was 0.060. Additional control for baseline health conditions reduced the coefficient slightly to 0.055, while community fixed effects yielded an estimate of 0.052. The result remained significant when standard errors were clustered. The negative control outcome showed a small and statistically non-significant coefficient of 0.006, which provides some reassurance that the observed association was not simply driven by generalized response bias (see Figure 6).

**Figure 6:**
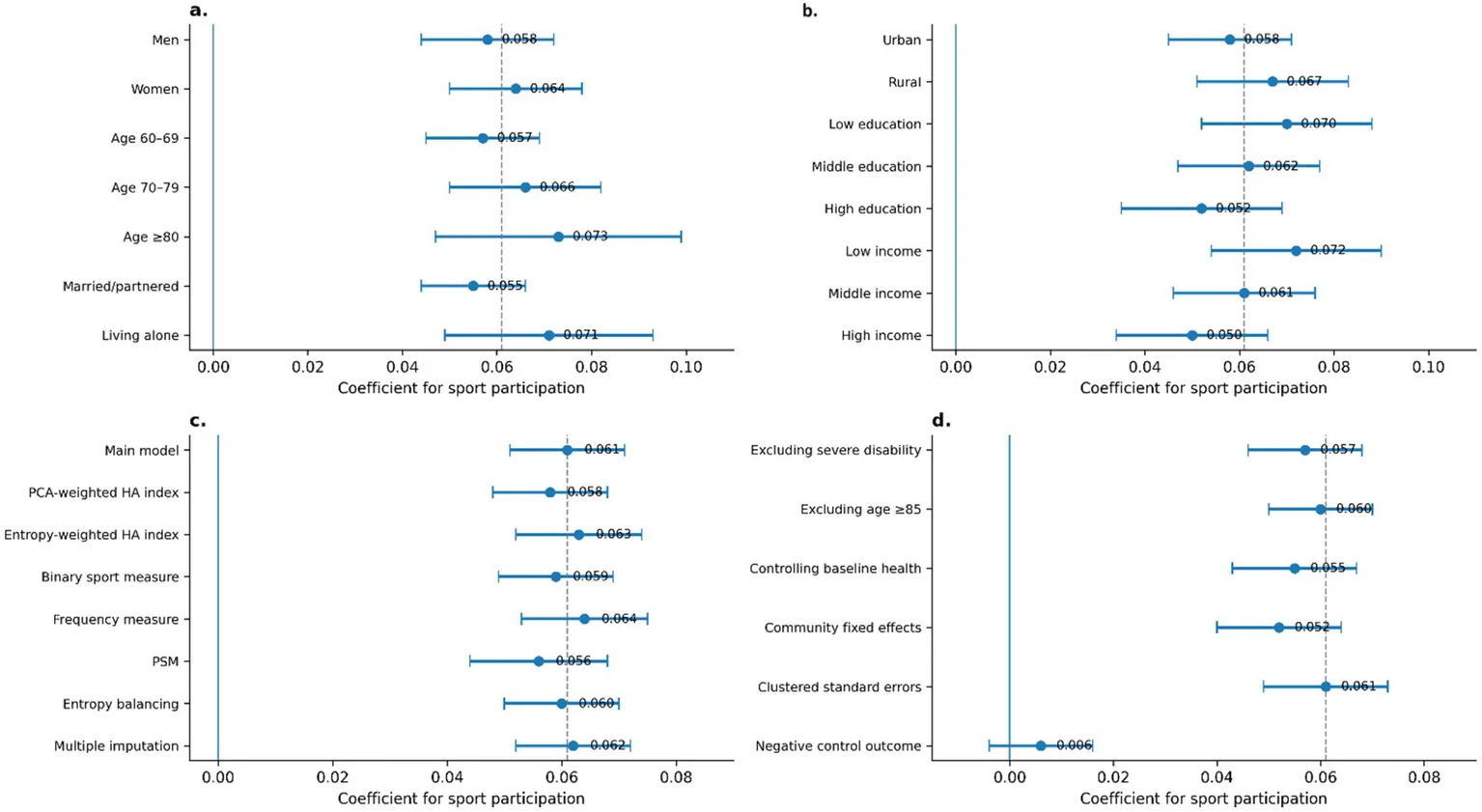
Heterogeneity and robustness of the association between sport participation and healthy aging. **(a)** Subgroup estimates by gender, age, and living arrangement. **(b)** Subgroup estimates by residence, education, and household income. **(c)** Robustness tests using alternative healthy aging indices, alternative sport participation measures, propensity score matching, entropy balancing, and multiple imputation. **(d)** Sensitivity analyses excluding vulnerable subgroups, strengthening confounding control, and using a negative control outcome. **(a)** Demographic heterogeneity. **(b)** Socioeconomic and residential heterogeneity. **(c)** Robustness across alternative specifications. **(d)** Sensitivity analyses.

These analyses indicate that the relationship between sport participation and healthy aging was robust across subgroup definitions, alternative variable construction and sensitivity specifications. The findings were strongest among older adults who were older, living alone, rural, less educated or lower-income, suggesting that sport participation may have particular public health relevance for groups facing greater aging-related vulnerability.

### 3.7 Empirical identification of vulnerable subgroups and differentiated benefit patterns

To move beyond average effects, subgroup analyses were conducted to identify populations showing different associations between sport participation and healthy aging. The results indicated that the association between sport participation and healthy aging varied across demographic, socioeconomic and functional characteristics.

Older-old adults aged 80 years and above showed a larger estimated association compared with younger older adults. The coefficient increased from 0.057 among adults aged 60–69 years to 0.073 among those aged 80 years and above. Similarly, older adults living alone showed a stronger association than those living with partners, with coefficients of 0.071 and 0.055, respectively. These findings suggest that groups with greater risks of functional decline or social isolation may obtain relatively larger benefits from sustained sport participation.

Socioeconomic differences also influenced the magnitude of association. Rural residents, individuals with lower education and those with lower household income showed stronger estimated associations compared with their more advantaged counterparts. However, these groups also demonstrated lower participation rates, indicating that greater potential benefits coexist with greater access barriers. This pattern highlights the importance of improving participation opportunities among vulnerable populations.

The dose–response analysis further provided empirical support for differentiated participation strategies. Healthy aging scores increased progressively from non-participation to moderate and frequent participation groups. However, the difference between high-volume participation and moderate-volume participation was relatively limited, suggesting that excessive increases in activity volume may generate diminishing returns. This pattern supports the importance of sustainable and appropriate participation rather than simply maximizing exercise quantity.

### 3.8 Robustness of the association under alternative identification strategies

The additional identification analyses supported the stability of the association between sport participation and healthy aging. After propensity score matching, the estimated coefficient remained positive at 0.056, which was close to the coefficient from the fully adjusted regression model. Entropy balancing produced a coefficient of 0.060, while doubly robust estimation yielded an estimate of 0.059. The consistency across different estimation strategies indicates that the relationship was unlikely to be entirely driven by observable differences between sport participants and non-participants. Balance diagnostics showed substantial improvement after matching and weighting. Standardized mean differences for demographic, socioeconomic, health-related and contextual variables were reduced below commonly accepted thresholds, indicating that the comparison groups became more comparable after adjustment. Sensitivity analyses also produced similar results. Excluding older adults with severe functional limitations reduced the coefficient slightly but maintained statistical significance. Additional adjustment for baseline health status and community fixed effects produced comparable estimates. These findings suggest that the observed association between sport participation and healthy aging remained robust under stricter identification conditions.

## 4 Discussion

The association remained after adjustment for demographic characteristics, socioeconomic status, health conditions and ecological context, and it showed a clear dose–response pattern. The result suggests that sport participation can be understood as a practical and measurable entry point for healthy aging interventions. Its value lies in its connection with several domains of later-life health, including physical function, emotional status, cognitive maintenance, social engagement and daily independence (30). The adjusted effect size should be interpreted in a realistic way. Sport participation did not explain healthy aging by itself, and the observed association was partly shaped by education, income, baseline health and community resources. However, the persistence of the association across models indicates that regular sport participation may contribute additional health advantages beyond pre-existing social and health conditions (31). This is important for public health practice because sport participation can be modified through community programs, environmental design and low-cost behavioral support.

The pathway analysis provides a more detailed explanation of how sport participation may contribute to healthy aging. Physical function was the strongest mediating pathway, which is consistent with the role of movement in maintaining strength, balance, mobility and endurance. For older adults, these capacities are directly related to daily functioning and the ability to remain independent. The finding suggests that the health meaning of sport participation should be evaluated not only by disease prevention, but also by whether it helps older adults preserve usable functional capacity. Social support and psychological resources also accounted for meaningful portions of the association. This finding is particularly relevant in the context of later life, where loneliness, bereavement, retirement and reduced social roles may gradually weaken everyday social contact (32). Many sport activities among older adults are group-based or community-based, such as walking groups, tai Chi-square dancing and fitness teams. These activities create repeated interaction, shared routines and a sense of belonging. Such social processes may help explain why sport participation was associated with better psychological well-being and broader healthy aging outcomes. The community activity environment had a smaller indirect effect but remained theoretically important. Its role may be more distal than physical function or social support. Safe spaces, accessible facilities and organized community activities may not directly improve health in a biological sense, but they shape the conditions under which older adults can participate regularly and safely. This finding reinforces the need to examine sport participation as a behavior embedded in place, service supply and neighborhood organization.

The moderation results showed that the association between sport participation and healthy aging was stronger in communities with better sports facility accessibility, safer neighborhoods, more walkable environments and stronger public service availability. This finding has direct implications for community health governance. Encouraging older adults to exercise is unlikely to be effective when the local environment lacks safe paths, nearby activity spaces, lighting, seating, barrier-free access or organized support (33). The same amount of motivation may lead to different health outcomes depending on whether the environment allows older adults to turn intention into sustained practice. The moderating role of facilities and walkability deserves particular attention. Older adults are more sensitive to environmental barriers than younger adults because mobility limitation, fear of falling and chronic disease symptoms may reduce their tolerance for unsafe or inconvenient spaces. A small distance, uneven pavement or lack of resting places may be enough to prevent regular participation. Therefore, the construction of age-friendly sport environments should emphasize proximity, safety, accessibility and low cost, rather than only increasing the number of facilities. Public service availability also strengthened the association, although the effect was slightly weaker. This may reflect the fact that services influence participation through organization, information and continuity. Community health centers, resident committees, public sports instructors and older-adult associations can help match activity types to health status, reduce uncertainty about exercise safety and maintain participation through group routines. These forms of support are especially relevant for older adults with chronic diseases or low exercise confidence.

The heterogeneity analysis showed stronger associations among rural residents, low-income older adults, less educated groups, older-old adults and those living alone. This pattern suggests that sport participation may generate larger marginal benefits among groups facing greater vulnerability. For these groups, sport participation may provide scarce opportunities for social contact, structured daily routines and functional maintenance. In rural or low-resource settings, even simple and regular activities such as walking, stretching, tai chi or community dancing may have meaningful value when formal health and social services are limited. At the same time, the descriptive results showed that disadvantaged groups were less likely to participate in sport. This creates a policy tension. Groups that may benefit more from sport participation often face greater barriers to accessing it. Low income, low education, chronic disease, weak family support and limited community infrastructure may all reduce participation. Therefore, public health interventions should not rely only on general health education or individual persuasion. More targeted support is needed for rural communities, older adults living alone, high-age groups and those with limited socioeconomic resources. A health equity perspective also requires attention to the quality and suitability of sport programs. Programs designed for relatively healthy and mobile retirees may exclude frailer older adults. Activities for vulnerable groups should be low-risk, progressive and adaptable. Community-level screening, simple exercise guidance, fall-prevention elements and links with chronic disease management can make sport participation more inclusive and safer.

The findings suggest several practical directions. Community sport promotion for older adults should be integrated with primary health care, chronic disease management and age-friendly community construction. Rather than treating physical activity promotion as a separate campaign, local public health systems can embed it into routine community health services, health education, follow-up visits and social participation programs. Facility planning should prioritize everyday accessibility. Small parks, walking paths, open squares, indoor activity rooms and age-friendly fitness stations may be more useful for many older adults than large, centralized sports venues. Environmental improvement should also include lighting, benches, toilets, non-slip surfaces, ramps and clear signage. These details determine whether older adults can participate without excessive risk or dependence on family accompaniment. Program design should emphasize continuity. Older adults are more likely to benefit from activities that are stable, familiar and socially supported. Community walking groups, tai chi classes, dancing teams, table tennis clubs and guided stretching sessions can create predictable participation routines. Public sports instructors, community doctors and volunteers may work together to provide basic safety guidance, especially for older adults with hypertension, diabetes, arthritis or cardiovascular risk.

The validation analysis strengthens confidence in the comparability of the healthy aging index across urban and rural populations. In aging research, indicators such as social participation, functional independence and psychological well-being may be influenced by contextual conditions. For example, rural older adults may rely more heavily on informal family networks, while urban older adults may have greater access to formal community services. Without testing measurement equivalence, direct comparisons may risk confusing contextual differences with differences in underlying health capacity. The findings indicate that healthy aging represents a relatively stable multidimensional construct across residential contexts, while the pathways supporting healthy aging remain context-dependent. This distinction is important for interpreting urban–rural differences. The same level of healthy aging may be achieved through different combinations of personal resources, social relationships and environmental support. Future research should continue to integrate contextual measurement with individual-level health indicators to better capture the complexity of aging experiences in diverse communities.

For older adults with relatively preserved functional capacity and adequate community resources, regular community-based activities such as walking groups, Tai Chi, recreational sports and organized fitness programs may help maintain physical function and social engagement. For older adults with functional limitations or chronic disease burdens, participation strategies should emphasize gradual progression, safety monitoring and adaptation to individual conditions. Low-intensity activities, supervised exercise and socially supported programs may provide more suitable entry points for maintaining activity continuity.

The observed dose–response pattern also provides important guidance for intervention design. The increase in healthy aging was most evident when older adults moved from inactivity to regular participation, whereas additional benefits appeared smaller at very high activity levels. This suggests that intervention priorities should focus on reducing inactivity and improving participation accessibility among inactive older adults. The establishment of safe exercise thresholds, however, requires evidence from longitudinal studies and intervention trials because the current observational design cannot determine causal dose–response boundaries.

From a practical perspective, intervention stratification should therefore be understood as a matching framework between individual capacity, activity characteristics and ecological support. Effective programs require coordination among older adults, families, community organizations and public health services. Such an approach can improve participation feasibility while reducing the risk that standardized exercise recommendations fail to accommodate diverse aging trajectories.

This study has several limitations. Although the present study provides evidence for heterogeneous associations between sport participation and healthy aging, several issues remain unresolved. The current data allow identification of groups with different benefit patterns but cannot establish precise safety thresholds or optimal exercise prescriptions for specific populations. The classification of activity intensity and risk levels was based on observational measures rather than experimental manipulation. Future research should combine longitudinal follow-up, wearable monitoring and randomized community interventions to determine the causal effects, safety boundaries and individualized exercise strategies for different older adult groups. Future research should use longitudinal data, wearable activity measures, community intervention trials or quasi-experimental designs based on facility construction and public sports program rollout. Further work should also distinguish different forms of sport participation, such as walking, tai chi, square dancing, resistance training and supervised exercise, and examine their suitability for frail older adults, rural residents, older women living alone and people with multimorbidity.

## Data Availability

The original contributions presented in the study are included in the article/supplementary material, further inquiries can be directed to the corresponding authors.
